# Early identification of lung cancer patients with venous thromboembolism: development and validation of a risk prediction model

**DOI:** 10.1186/s12959-023-00544-w

**Published:** 2023-09-14

**Authors:** Wenjuan Di, Haotian Xu, Chunhua Ling, Ting Xue

**Affiliations:** 1 Department of Pulmonary and Critical Care Medicine, Kunshan Hospital of Traditional Chinese Medicine, Suzhou City, Jiangsu Province People’s Republic of China; 2 Department of Pulmonary and Critical Care Medicine, The First Hospital Affiliated of Soochow Unversity, No188, Shizi Street, Gusu district, Suzhou City, Jiangsu Province People’s Republic of China

**Keywords:** Lung cancer, Venous thromboembolism, Biomarker, Risk factor, Risk prediction model

## Abstract

**Introduction:**

Venous thromboembolism(VTE) is a leading cause of death in patients with lung cancer. Furthermore, hospitalization of patients with advanced lung cancer for VTE treatment represents a major economic burden on the national public health resources. Therefore, we performed this prospective study to identify clinical biomarkers for the early identification of VTE in lung cancer patients.

**Methods:**

This prospective study enrolled 158 patients with confirmed lung cancer, including 27 who were diagnosed with VTE within six months of the follow-up after lung cancer diagnosis. Multivariate logistic regression analysis was used to evaluate the diagnostic performancese of all the relevant clinical features and laboratory indicators in identifying lung cancer patients with a higher risk of VTE. A novel risk prediction model was constructed consisting of five clinical variables with the best diagnostic performances and was validated using the receiver operation characteristic(ROC) curves. The diagnostic performances of the new risk prediction model was also compared with the Khorana risk score (KRS) and the Padua risk score (PRS).

**Results:**

The VTE group of lung cancer patients (n = 27) showed significantly higher serum levels of fibrin degradation products (FDP), D-dimer, thrombomodulin (TM), thrombin-antithrombin-complex (TAT), α2-plasmin inhibitor-plasmin Complex (PIC), and tissue plasminogen activator-plasminogen activator inhibitor complex (t-PAIC) compared to those in the non-VTE group (n = 131). ROC curve analyses showed that the diagnostic efficacy of the new VTE risk prediction model with TM ≥ 9.75 TU/ml, TAT ≥ 2.25ng/ml, t-PAIC ≥ 7.35ng/ml, history of VTE, and ECOG PS score ≥ 2 was superior than the KRS and the PRS in the early identification of lung cancer patients with a higher risk of VTE.

**Conclusions:**

The new risk prediction model showed significantly high diagnostic efficacy in the early identification of lung cancer patients with a high risk of VTE. The diagnostic efficacy of the new risk prediction model was higher than the KRS and the PRS in this cohort of lung cancer patients.

## Introduction

In recent years, the prognostic estimation of lung cancer to guide therapeutic strategy is not limited to developmental status and the genetic nature of the tumors, but also takes into account the effects of concomitant complications. Venous thromboembolism (VTE) is a multifactorial disease that includes deep vein thrombosis (DVT) and pulmonary embolism (PE) and is the most common complication associated with lung cancer [1]. Previous epidemiological surveys have shown that the incidence of cancer-associated thrombosis (CAT) is 4-20% in the lung cancer patients [2]. In comparison with gastrointestinal cancer, the relative risk of VTE is lower in patients with lung cancer but the absolute number of VTE events are higher because of increased prevalence of lung cancer [3]. In recent years, significant advances in medical care have improved the treatment options and the survival rates of cancer patients. The increasing incidence of CAT over time is influenced by the tumor types and stages, treatment-associated factors, and patient-related risk factors. In the lung cancer patients, vasular endothelial injury caused by the use of chemotherapy, targeted therapy, or immunotherapy significantly increased the rate of incidental thrombosis; moreover, bone marrow suppression after chemotherapy increased the risk of CAT [4]. The incidence of CAT is associated with the increased morbidity and mortality rates of lung cancer patients. According to the 2019 American Society of Clinical Oncology (ACSO) guidelines, many risk factors need to be considered before considering the type of anticoagulant therapy for treating CAT in the cancer patients [5]. Only few hospitalized cancer patients with acute medical illness require thromboprophylaxis, which is not routinely recommended for the cancer patients. Anticoagulant therapy can cause, major bleeding complications or hemorrhages that can be fatal. The Costecat study reported significant economic burden for both the lung cancer patients and the public healthcare systems due to VTE episodes in the lung cancer patiens with hospitalizations accounting for 65.8% of the total costs [6].

Therefore, early diagnosis and treatment of VTE is necessary for improving the survival rates of patients with lung cancer. Several risk prediction models have been established based on observational or prospective studies for the early identification of cancer patients with a higher risk of VTE. Khorana risk score (KRS) [7] is the most commonly used risk prediction model for stratifying cancer patients into different risk levels based on five baseline clinical and laboratory variables to identify the high-risk group for thromboprophylaxis. However, Mansfield et al. [8] reported that high KRS was not associated with VTE but was an independent predictor of all-cause mortality in the lung cancer patients. In a prospective study in Korea [9], the Padua score was used to assess the incidence of VTE within 14 days of hospitalization in elderly cancer patients. Therefore, a validated risk prediction model is not available for accurately predicting VTE in the lung cancer patients. Furthermore, recent studies have shown that several cancer therapeutic regimens are associated with an increased risk of thromboembolic diseases [10]. Frustratedly, it does not seem to apply to cancer patients in China. Therefore, a new risk prediction score that considers both genetic and therapeutic factors is required to accurately predict the risk of VTE in the lung cancer patients.

In this prospective study, we investigated the risk factors associated with VTE in the hospitalized lung cancer patients. Furthermore, we aimed to identify potential risk factors by analyzing the association of thrombosis in the lung cancer patients with clinical features such as age, gender, cancer site, histopathological type, tumor stage, history of VTE, obesity, and ECOG PS score, and laboratory indicators such as FDP, D-dimer, TM, TAT, PIC, and t-PAIC. We then constructed and validated a new clinical risk prediction model for the early identification of lung cancer patients with a high risk of VTE. Finally, the diagnostic efficacy of the new prediction model in detecting VTE was compared with the KRS and the PRS in the lung cancer patients.

## Methods

### Study population

This prospective single-center study enrolled patients over 18 years old with cytologically or histologically confirmed lung cancer that were admitted to the First Affiliated Hospital of Soochow University between October 2019 and February 2021. The staging of lung cancer was based on the Eighth edition of the International TNM staging criteria for lung cancer developed by the International Association for the Study of Lung Cancer (IASLC) [10]. The exclusion criteria included patients with prophylactic anticoagulation, pregnancy, and those using anticoagulants within three months prior to study inclusion. This study was approved by the Ethics Committee of the First Affiliated Hospital of Soochow University. We obtained signed informed consent forms from all of the enrolled patients.

### Study protocol

This study enrolled 158 patients who met the inclusion criteria in this single-center study. The electronic medical records of the study subjects were obtained from the hospital archives. The patients received regular therapy regimens according to the guidelines. The primary end point of this study was confirmation of VTE in the asymptomatic patients based on the computed tomography pulmonary angiography (CTPA) and the lower extremity vascular ultrasound. The following factors were extracted from the medical records to facilitate diagnosis and treatment: gender, age, histopathological type, tumor stage and distant metastasis, complications, body mass index (BMI), time of initial diagnosis of lung cancer, ECOG PS score, and laboratory indicators. The aim was to identify the risk factors that were associated with the early identification of lung cancer-associated thrombosis.

### VTE diagnosis

The patients enrolled in this study were examined by computed tomography and color Doppler ultrasound of both the lower limbs to confirm or exclude VTE every time they were hospitalized. Patients with symptoms of PE underwent CT pulmonary angiography (CTPA) for the confirmatory diagnosis. Each thrombotic event was analyzed by an independent panel of experts in angiography, radiology, and clinical medicine to evaluate and confirm the event. The results were not affected by the slight gap caused by different personnel of the expert group.

### Data collection

The clinical and laboratory data of all patients was obtained from the electronic medical records. The laboratory variables included coagulation-related biomarkers (FDP and D-dimer) and thrombus-related biomarkers (TM, TAT, PIC, and t-PAIC). Thrombus-related biomarkers comprehensively evaluate the vascular endothelial injury and the activation of thrombin and plasmin. Thus, the combined detection of thrombus-related molecular biomarkers, d-dimer, and FDP represented a more sensitive and reliable estimation of the occurrence and formation of thrombus in the initial stages.

### Estimation of the laboratory parameters

Fasting elbow venous blood of all the study participants was collected in the morning and analyzed using the automatic chemiluminescence immunoassay with the matching chemiluminescence reagent (Sysmex, HISCl-800) and the automatic coagulation analyzer (Sysmex, CS2000i). (1) Automatic chemiluminescence immunoassay: We collected 2 ml blood in an anticoagulant citrate tube and performed the automatic chemiluminescence immunoassay with the matching chemiluminescence reagent to detect the levels of TM, TAT, PIC, and tPAI-C. (2) Automatic coagulation analyzer: We collected 1.8 ml blood in the sodium citrate anticoagulant tube, and the levels of FDP and D-dimer were determined using the automatic coagulation analyzer.

### Statistical analysis

At the end-point, the enrolled patients (n = 158) were assigned into the following two groups: (1) VTE group (17.1%, 27 patients) and (2) non-VTE group (82.9%, 131 patients). The consecutive variables with normal distributions were expressed as means ± standard deviation (SD) and those with abnormal distributions were expressed as medians (interquartile range). The statistical data was analyzed using the IBM SPSS Statistics for Windows (IBM Corp. Version 26.0. Armonk, NY, USA). The clinical characteristics of enrolled patients were expressed as frequencies and percentages for all the categorical variables. The count data between groups were compared using the chi-square test. The measurement data of the continuous variables were compared using the t test or the Mann-Whitney U test. P < 0.05 was considered as statistically significant. Multivariable logistic regression analysis was used to identify the independent risk factors associated with lung cancer-associated thrombosis. Furthermore, the independent risk factors were combined to construct a new risk prediction model using R programming [11]. The diagnostic performances of the new risk prediction model was compared with the KRS and the PRS using our study cohort.

## Results

### Comparison of baseline clinical characteristics between lung cancer patients with or without VTE

This study enrolled 158 participants that met the inclusion criteria. Thrombotic events were reported in 27 (17.1%) patients (VTE group) within six months after the diagnosis of lung cancer. The baseline clinical characteristics of the VTE group and the non-VTE group patients are summarized in Table 1. Overall, 76.6% (n = 121) of the study participants were male. There were no significant differences in the gender distribution between the two groups. The median age of patients diagnosed with thrombosis was 71.41 years, which was comparatively higher than the median age of patients in the non-thrombosis group (64.36 years). Furthermore, 29.6% (n = 8) of the patients in the VTE group showed a BMI ≥ 25 kg/m². Therefore, the median BMI of the VTE group was significantly higher than the BMI of patients in the non-VTE group. Lung cancer patients with advanced stage or distant metastases were more likely to develop VTE than those with localized lesions (92.6% vs. 67.9%, P = 0.009; Table 1). Furthermore, the number of patients with ECOG PS scores ≥ 2 were significantly higher in the VTE group compared to the non-VTE group (81.5% vs. 21.7%, p < 0.001). Besides, history of VTE was an important risk factor of lung cancer-associated thrombosis. The number of patients with history of VTE were significantly higher in the VTE group compared to the non-VTE group (37.0% vs. 4.6%, p < 0.001). Moreover, we did not observe significant differences in the incidence rates of thrombotic events between patients with non-small cell lung cancer (NSCLC) and those with small cell lung cancer (SCLC). However, the incidence rates of thrombotic events were significantly higher in the lung cancer patients with hypertension compared to those without hypertension (63.0% vs. 36.6%) in our study.

**Table 1:** Characteristics between the VTE group and the non-VTE group

Group	n	Gender (M/F)	Age ($\stackrel{-}{x}$±s)	ECOG PS (< 2/≥2)	History of VTE (Yes/No)	NSCLC/SCLC	Clinical stage (III-IV/I-II)	Hypertension (Yes/No)	Atrial fibrillation (Yes/No)	BMI (≥ 25/<25)
VTE	27	14/13 (51.9%)	71.41 ± 8.16	5/22 (18.5%)	10/17 (37.0%)	22/5 (81.5%)	25/2 (92.6%)	17/10 (63.0%)	5/22 (18.5%)	8/19 (29.6%)
Non-VTE	131	102/29 (77.9%)	64.36 ± 9.90	96/35 (73.3%)	6/125 (4.6%)	112/19 (85.5%)	89/42 (67.9%)	48/83 (36.6%)	3/128 (2.3%)	15/116 (11.5%)
χ²/t		0.701	3.722	29.113	25.912	0.280	6.772	6.405	12.265	5.948
P-value		0.403	0.054	< 0.001*	< 0.001*	0.597	0.009*	0.011*	< 0.001*	0.015*

Abbreviation:* p-Value < 0.05

### Identification of serum biomarkers associated with VTE

We then analyzed the relationship between thrombotic events and the coagulation-and thrombus-related biomarkers to identify the biomarkers associated with early diagnosis of thrombosis. Firstly, the Kolmogorov-Smirnov test results showed that all the continuous variables analyzed did not conform to normal distribution. Therefore, they were represented as median (interquartile range). The Mann-Whitney U test was used to analyze the differences in these variables between the VTE and the non-VTE groups. The serum levels of FDP, D-dimer, TM, TAT, PIC, and t-PAIC were significantly higher in the VTE group compared with the non-VTE group (P < 0.05) (Table 2).

**Table 2:** Comparison between the VTE group and the non-VTE group.(M [Q1,Q3]).

Group	n	FDP(mg/L)	D-dimer(µg/mL)	TM(TU/ml)	TAT(ng/ml)	PIC(ug/ml)	tPAI-C(ng/ml)
VTE	27	11.01(4.40-28.07)	3.52(1.27–9.49)	12.60(10.70–15.10)	4.50(2.70–9.50)	1.64(1.03–1.95)	8.90(7.60–14.70)
Non-VTE	131	2.59(1.77–4.28)	0.58(0.37–1.27)	9.30(7.70–10.70)	2.20(1.30–3.30)	0.95(0.70–1.39)	7.10(5.10–9.10)
Z value		-6.015	-5.871	-5.142	-4.870	-3.989	-3.005
P-value		< 0.001*	< 0.001*	< 0.001*	< 0.001*	< 0.001*	= 0.003*

Abbreviation:* p-Value < 0.05

### Receiver Operating Curve (ROC) analysis of the diagnostic efficacy of the VTE-related biomarkers in the cohorts of lung cancer patients

**Fig. 1:**
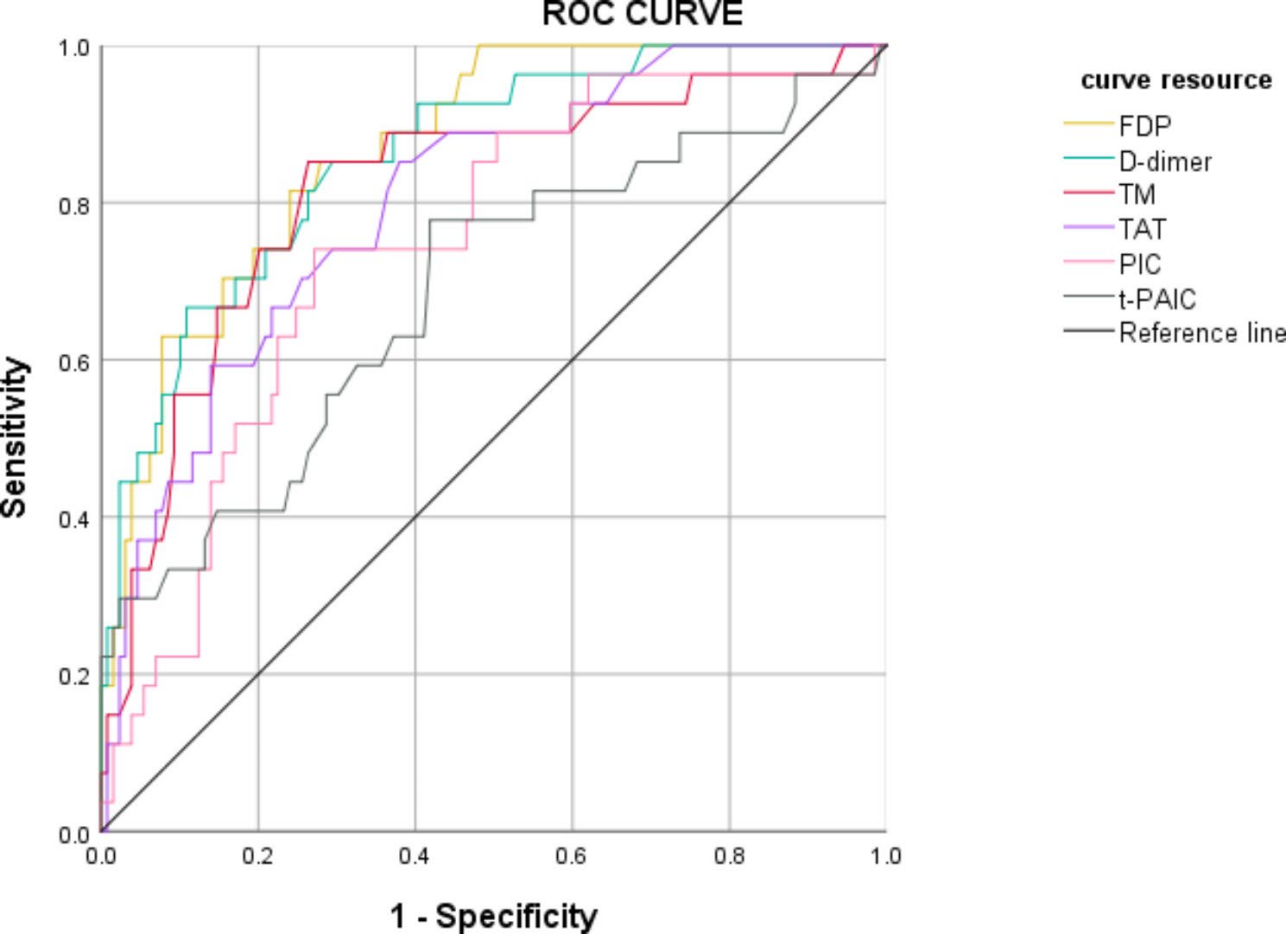
Receiver operating characteristic (ROC) curve analysis of each biomarker in diagnosis of VTE.

**Fig. 2:**
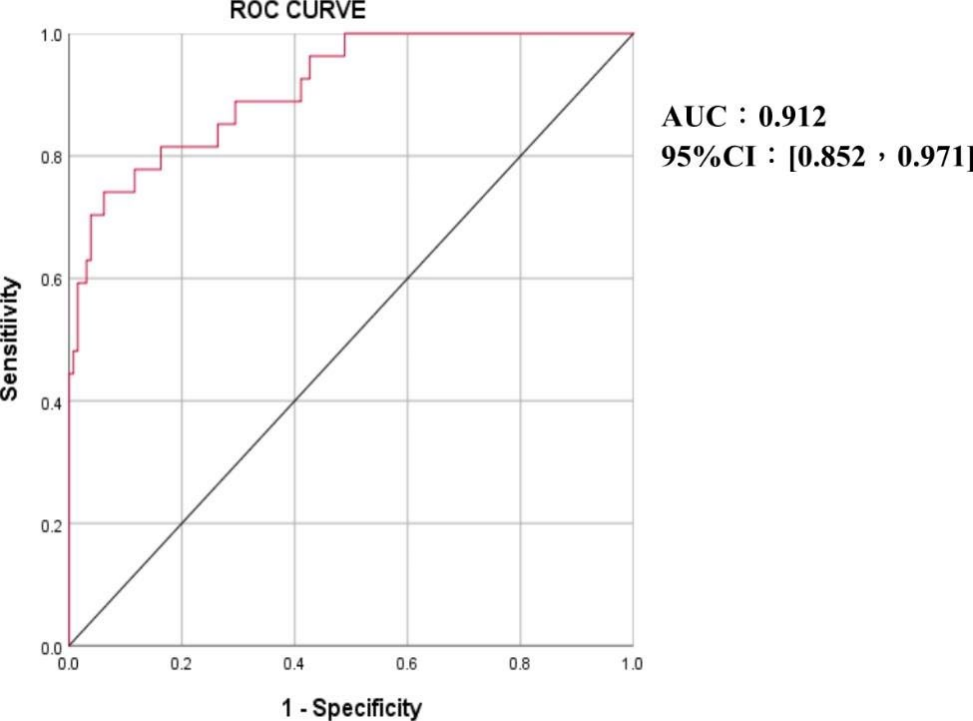
Receiver operating characteristic (ROC) curve analysis of combined 6 biomarkers in diagnosis of VTE.

The area under the curve (AUC) values of FDP, D-dimer, TM, TAT, PIC, and t-PAIC for diagnosing VTE were 0.869, 0.860, 0.817, 0.802, 0.745, and 0.685, respectively. The cut-off values for FDP, D-dimer, TM, TAT, PIC, and t-PAIC were 2.84 mg/L, 0.77 µg/mL, 9.75 TU/ml, 2.25 ng/ml, 0.80 µg/ml, and 7.35 ng/ml, respectively(Table 3; Fig. 1). The sensitivity values of FDP, D-dimer, TM, TAT, PIC, and t-PAIC for the diagnosis of VTE were 96.3%, 92.6%, 88.9%, 88.9%, 92.6% and 77.8%, respectively. The specificity values of FDP, D-dimer, TM, TAT, PIC, and t-PAIC for the diagnosis of VTE were 45.7%, 40.3%, 40.3%, 48.1%, 60.5%, and 45.0%, respectively. Furthermore, we found that the combined diagnostic efficacy of the six biomarkers was higher than the diagnostic efficacy of the individual biomarkers (AUC value of 0.912) (Fig. 2)

**Table 3:** Diagnostic efficiency of each biomarker in lung cancer-associated VTE.

Variable	AUC	Cut-off value	Sensitivity	Specificity	Youden index	95%CI
FDP	0.869	2.84	0.963	0.457	0.506	0.803–0.934
D-dimer	0.860	0.77	0.926	0.403	0.523	0.786–0.934
TM	0.817	9.75	0.889	0.403	0.486	0.723–0.911
TAT	0.802	2.25	0.889	0.481	0.408	0.716–0.889
PIC	0.745	0.80	0.926	0.605	0.321	0.647–0.843
t-PAIC	0.685	7.35	0.778	0.450	0.328	0.566–0.805

### Selection of the valuable variables for building new model by multivariable identification of VTE-associated risk factors using logistic multiple regression analysis

We analyzed the results of the Chi-square test and the sample sizes, and identified the following factors as candidated risk factor for further screening: clinical stage, hypertension, BMI ≥ 25 Kg/m², ECOG PS score ≥ 2, history of VTE, FDP ≥ 2.84 mg/L, D-dimer ≥ 0.77 µg/g/ml, TM ≥ 9.75 TU/ml, TAT ≥ 2.25 ng/ml, PIC ≥ 0.80 µg/ml, and tPAI-C ≥ 7.35 ng/ml. Subsequently, the collinearity of these factors was analyzed. The results showed multicollinearity between numerous clinical factors with FDP and D-dimer (Table 4). The high correlation between the coagulation-related biomarkers and the thrombus-related biomarkers reduced the accuracy of the risk prediction model. Therefore, we excluded the aforementioned non-compliance indicators, such as clinical stage, hypertension, BMI ≥ 25 Kg/m², and PIC. The remaining indicators were used as covariates and multivariate logistic regression analysis was performed with the occurrence of VTE as the dependent variable. The results showed that TM ≥ 9.75 TU/ml (OR = 1.616, 95%CI:1.219–20.763, P = 0.026), TAT ≥ 2.25 ng/ml(OR = 2.480, 95%CI:2.401–59.386, P = 0.002), t-PAIC (OR = 1.578, 95%CI:1.301–18.055, P = 0.019), history of VTE (OR = 2.071, 95%CI:1.630-38.617, P = 0.010), and ECOG PS score ≥ 2 (OR = 2.208, 95%CI:2.536–32.616, P = 0.001) were independent risk factors for the lung cancer-associated thrombosis (Table 5).

**Table 4:** Results of multicollinearity analysis

Variables	Tolerance	VIF
Clinical stage	0.774	1.291
Hypertension	0.814	1.228
Obesity(BMI ≥ 25)	0.915	1.093
ECOG PS	0.720	1.389
History of VTE	0.846	1.182
FDP	0.092	10.862*
D-dimer	0.074	13.499*
TM	0.798	1.252
TAT	0.892	1.121
PIC	0.465	2.150
tPAI-C	0.665	1.505

Abbreviation:* The factor is multicollinearity

**Table 5:** Risk factors for thrombosis in lung cancer patients by multivariate analysis

Variable	B	SE	Wald	*P-value*	Oddsratio (OR)	OR 95% CI	
						lower	upper
TM ≥ 9.75TU/ml	1.616	0.723	4.989	0.026*	5.031	1.219	20.763
TAT ≥ 2.25ng/ml	2.480	0.818	9.182	0.002*	11.941	2.401	59.386
t-PAIC ≥ 7.35ng/ml	1.578	0.671	5.532	0.019*	4.846	1.301	18.055
History of VTE	2.071	0.807	6.581	0.010*	7.935	1.630	38.617
ECOG PS score ≥ 2	2.208	0.652	11.478	0.001*	9.094	2.536	32.616
Constant	-7.050	1.259	31.364	0.000	0.001		

Abbreviation:* p-Value < 0.05

### Construction and validation of a new risk prediction model for lung cancer-associated thrombosis

**Fig. 3:**
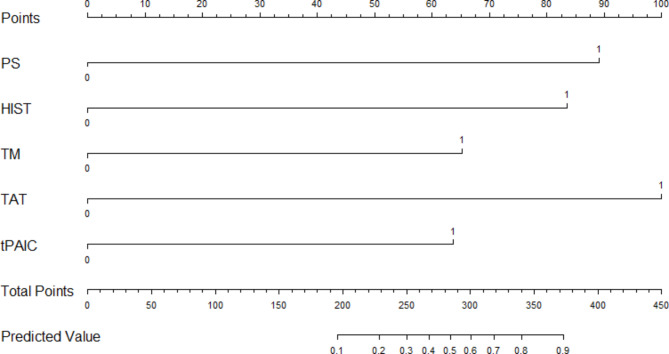
Nomogram of the new risk prediction model. (*Abbreviation:PS: ECOG PS score ≥ 2; HIST: History of VTE; TM: TM ≥ 9.75TU/ml; TAT: TAT ≥ 2.25ng/ml; t-PAIC: t-PAIC ≥ 7.35ng/mL*)

A new risk prediction model was constructed by incorporating the independent risk factors. The data was imported into the R software [11] and the rms package [12] was used to create a nomogram. Each predictor in the nomogram was assigned a score on the “Points” axis. The sum of scores for all the variables were assigned to the “Total Points” axis. The total points corresponded to the predicted probability of VTE associated with lung cancer(Fig. 3).

**Fig. 4:**
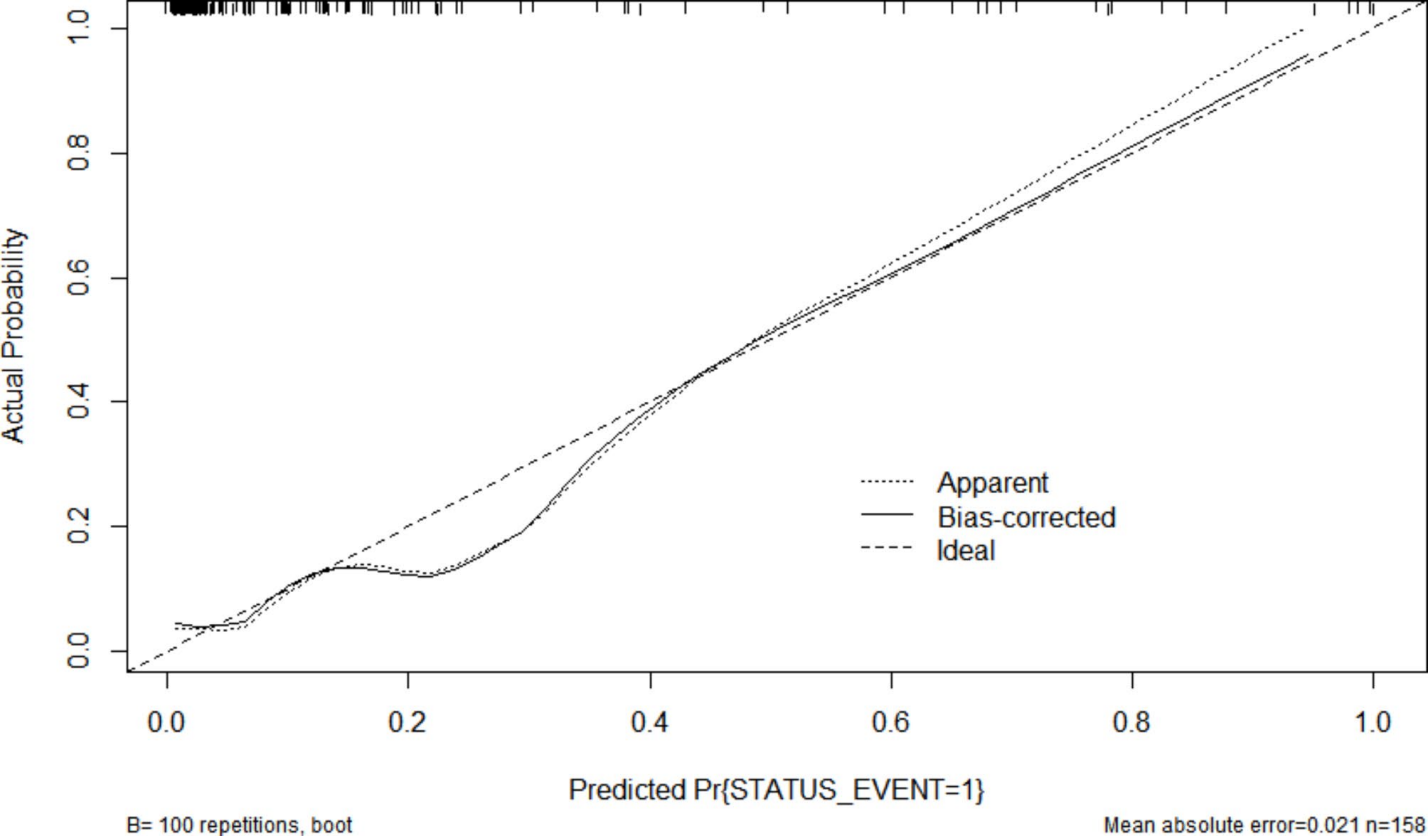
Calibration curve of the new model

The Omnibus test of model coefficients showed that the new VTE risk prediction model was significant (χ²=69.377, P < 0.001). The Hosmer and Lemeshow test indicated high goodness of fit test for the new VTE risk prediction model (P = 0.662 > 0.05). The C-statistic value for the discrimination ability of the new risk prediction model was 0.889 (95% CI: 0.829–0.933). The C-statistic value for the new VTE risk prediction model was 0.862 after internal validation using bootstrap resampling. The calibration plot showed good agreement between the calibration curve and the ideal curve, thereby suggesting good agreement between the predicted incidence rate of the model and the actual incidence rate (Fig. 4).

### Comparison of diagnostic efficacy between the new risk prediction model and the other two

**Fig. 5:**
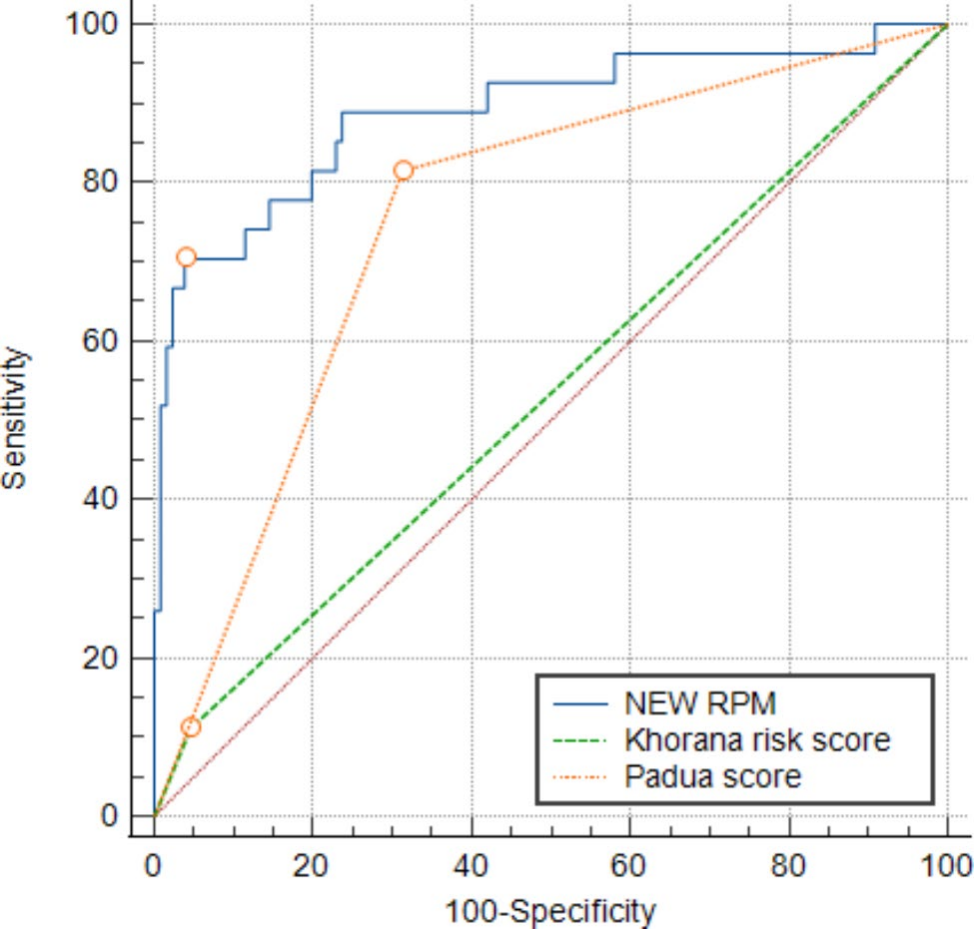
Comparison of C-statistic values. (*Abbreviation: NEW RPM:new risk prediction model,Padua score:Padua risk score*)

**Fig. 6:**
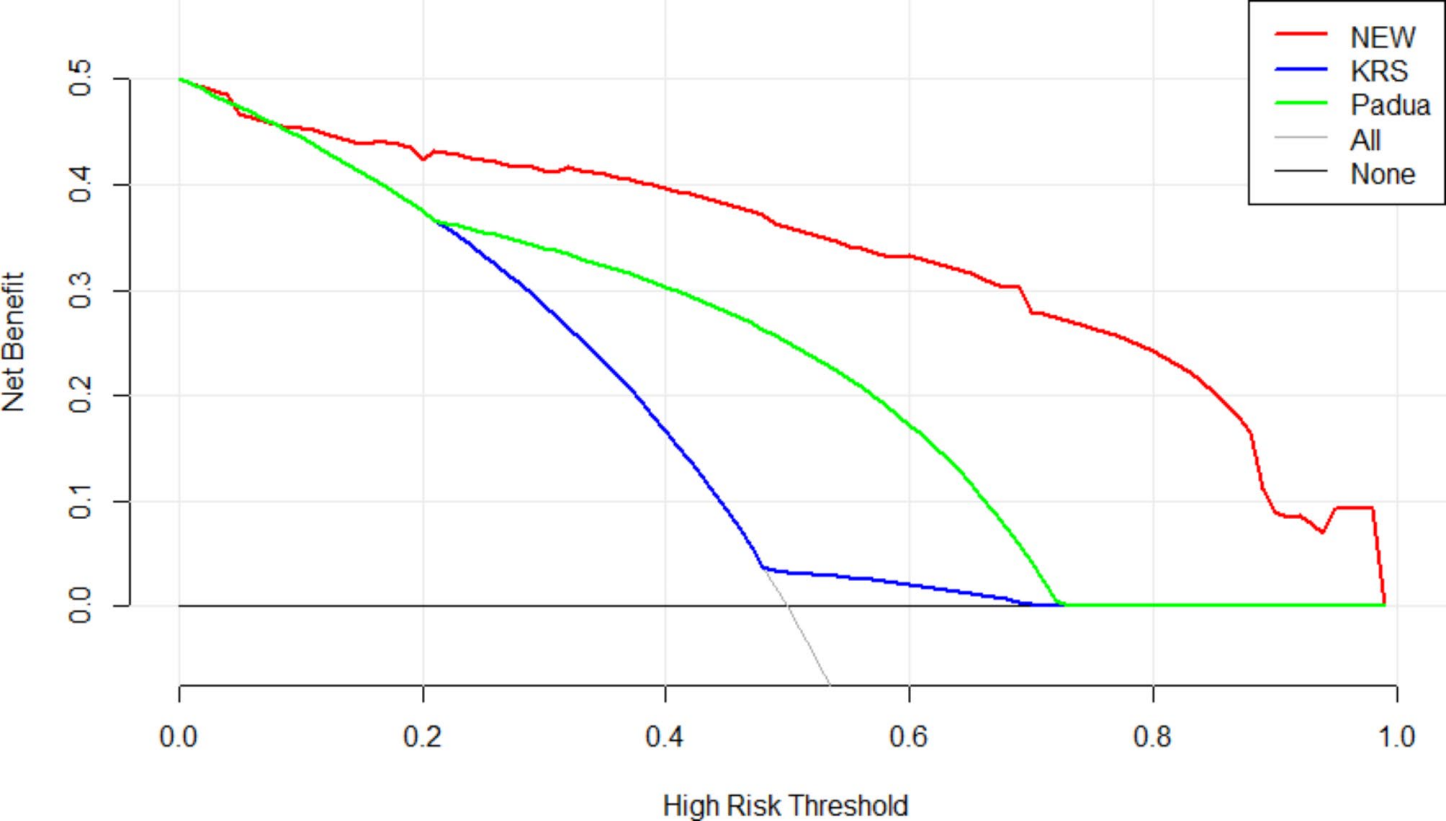
Comparison of Clinical decision curve analysis. (*Abbreviation: NEW:new risk prediction model,KRS:Khorana risk score,Padua:Padua risk score*)

Next, we compared the diagnostic efficacies of the new VTE prediction model with the KRS and the PRS. The − 2loglikelihood ratio of the new prediction model was 75.126, more effective than the KRS (139.674) and the PRS (96.188)(Table 6). The C-statistic values for the new VTE risk prediction model, the KRS, and the PRS were 0.889, 0.533, and 0.751 respectively (Fig. 5). This validated the higher diagnostic efficacy of the new VTE prediction model. Furthermore, we verified the clinical applicability of the new risk prediction model. Clinical decision curve analysis can visualize the clinical benefit of the model at different thresholds, and the results show that the new risk prediction model has a higher net benefit rate than the KRS and the PRS(Fig. 6).

**Table 6:** Comparison of C- statistics between the new RPM and the other two

Model	-2 LogLikelihood	C-statistic
the new RPM	75.126	0.889
the KRS	139.674	0.533
the PRS	96.188	0.751

*Abbreviation: RPM: risk prediction model,KRS:Khorana risk score,PRS:Padua risk score*

## Discussion

Previous studies have identified several clinical and genetic factors that may be associated with the risk of cancer-associated thrombosis. Previously, only patients with clinical symptoms such as chest pain, swelling of the extremities, and hemoptysis were analyzed for potential thrombotic events. Therefore, thrombosis was not detected in most lung cancer patients until after death or at autopsy. Advances in medical technology have increased the use of precision medicine by the clinicians for disease prevention. The incidence of lung cancer-associated thrombosis ranges from.

4–20%. [2] Thromboembolic disease plays a significant role in tumor progression and is the most common cause of death in patients with lung cancer. The treatment of thrombosis is controversial, especially in lung cancer patients, because it can cause fatal hemorrhagic events. Therefore, early detection and prevention of thrombosis is critical for increasing the survival rates of the lung cancer patients.

In this study, we focused on the risk factors of thrombosis, and identified high-risk lung cancer patients in our cohort to evaluate potential risk factors and laboratory indicators. The incidence rates of VTE events within six months of lung cancer diagnosis in our cohort was 17.1%. This was comparable with the previously reported findings. [2] [3] [4] The thrombosis-related factors were periodically monitored in all the lung cancer patients included in this study. Currently, imaging tests are considered as the gold standard for diagnosing VTE. However, plasma biomarkers offer immense clinical value for the detection of VTE because they are non-invasive, decrease the time of detection and eliminate the exposure to radiation. Although D-dimer is the most widely used biomarker for evaluating VTE, it is associated with high sensitivity and low specificity. [5] Therefore, timely and accurate diagnosis of VTE is an ongoing challenge for the lung cancer patients prone to thrombosis and monitoring of pre-coagulation or pre-fibrinolysis factors is critical for identifyin the thrombotic events. Hence, in our study, we analyzed a panel of 4 thrombus related-biomarkers, namely, TM, TAT, PIC, and tPAI-C, which were closely related to coagulation, fibrinolysis, and endothelial function. [13] Thrombomodulin(TM) is a glycoprotein on the surface of endothelial cells that forms a 1:1 complex with thrombin. The TM-thrombin complex activates protein C approximately 1,000 times higher than thrombin alone and contributes to effective inactivation of the coagulation factors Va and VIIIa. Therefore, TM is a useful biomarker of endothelial injury. [14] Thrombin is generated by the proteolytic activation of prothrombin and represents the first step of the thrombus formation pathway. Thrombin activates several downstream substrates involved in thrombus formation. Thrombin rapidly interacts with anti-thrombin (AT) to form the thrombin-antithrombin (TAT) complex, which is considered as a coagulation related-biomarker because its levels are elevated in patients with VTE. [15] Alpha-2 plasmin inhibitor complex (PIC) is an irreversible complex of the enzyme plasmin and its inhibitor α2-anti-plasmin. It is not detected in vivo. Besides, t-PAIC is a 1:1 covalent inactive complex of tissue plasminogen activator (tPA) and plasminogen activator inhibitor-1 (PAI-1), and represents an important marker of the fibrinolytic system in the diagnosis of VTE. [16]. Our data showed that all the thrombosis-related biomarkers were significantly elevated in the VTE group compared to the non-VTE group. These results were in full agreement with the results reported by Zhou et al. [17] ROC curve analysis showed that the AUC values of the thrombosis-related biomarkers were lower than the AUC values of the d-dimer and FDP, but their diagnostic specificity was higher. D-dimer and FDP were removed because of multicollinearity in the multivariate analysis.

The newly constructed risk prediction model in this study was composed of five indicators, namely, ECOG PS score ≥ 2, history of VTE, TM ≥ 9.75 TU/ml, TAT ≥ 2.25 ng/ml, and t-PAIC ≥ 7.35 ng/ml. A nomogram was constructed with these 5 indicators using the rms package of the R software. The results of the Omnibus test of model coefficients showed that the new risk prediction model was meaningful. The results of the Hosmer and Lemeshow test for model fitness, showed that sufficient information was extracted for the data analysis. The new VTE risk prediction model showed a high degree of fit. In comparison with the KRS and the PRS, the new risk prediction model showed higher diagnostic efficacy. Therefore, this new risk prediction model shows significant clinical value for identifying lung cancer patients with higher risk of VTE. However, further validation of the model is required for confirming our results.

Several clinical trials of different oral anticoagulants have been conducted with promising results. However, currently, clinicians prioritize treatment with low molecular weight heparin as an anticoagulant for patients with a risk of thrombosis. According to the 2019 ASCO clinical practice guidelines, [5] the use of parenteral anticoagulants is recommended for thromboprophylaxis in hospitalized cancer patients and in patients undergoing cancer surgery to greatly reduce the morbidity due to thrombosis. Currently, there is insufficient evidence, whether preventive anticoagulation increased the risk of fatal bleeding in patients with lung cancer. Therefore, stratification of patients can reduce the number of patients requiring treatment with anticoagulants. This can prevent the occurrence of venous thromboembolism in cancer patients and reduce the occurrence of bleeding events.

This study has several shortcomings. Firstly, this was a single center study with a small sample size. The incidence rate of VTE among the 158 lung cancer patients enrolled in this study was only 17.1%. Therefore, future studies with a larger sample size are necessary for further validating our findings. Furthermore, survival outcomes were not analyzed in this study because of a short follow-up period of six months. Secondly, the secretion of t-PAIC is time-dependent. [17] However, collection of blood specimens was based on the clinical need and may have affected the diagnostic efficacy. Finally, we did not further investigate the anticoagulation regimen in lung cancer patients with concomitant thrombosis. We also did not analyze the changes in coagulation-related biomarkers and thrombosis-related biomarkers after anticoagulation therapy. The treatment of thrombosis is also related to the cancer treatment regimen [18]. Therefore, in future studies, health condition of the cancer patients and the current chemotherapy regimen needs to be considered to avoid fatal bleeding events.

## Conclusion

In conclusion, this study demonstrated higher diagnostic efficacy of the new risk prediction model with five clinical indicators, namely, ECOG PS score ≥ 2, history of VTE, TM ≥ 9.75 TU/ml, TAT ≥ 2.25 ng/ml, and tPAI-C ≥ 7.35 ng/ml, compared to the KRS and the PRS for detecting the risk of VTE in a cohort of 158 lung cancer patients. This new VTE risk prediction model showed significant clinical value for the early identification of lung cancer patients with a higher risk of VTE. This new risk prediction model can be used to periodically monitor lung cancer patients with higher VTE risk and can decrease the mortality and morbidity rates due to lung cancer-associated VTE. The findings of our study need to be verified in the future with larger-cohort multi-center clinical studies.

## Data Availability

These data can all be queried in the case system of the First Affiliated Hospital of Soochow University.
